# Effect on Cellular Vitality In Vitro of Novel APRF-Chlorhexidine Treated Membranes

**DOI:** 10.3390/jfb13040226

**Published:** 2022-11-07

**Authors:** Tasho Gavrailov, Ivan Chenchev, Maria Gevezova, Milena Draganova, Victoria Sarafian

**Affiliations:** 1 Department of Oral Surgery, Medical University—Plovdiv, 4000 Plovdiv, Bulgaria; 2 Research Institute at MU-Plovdiv, 4000 Plovdiv, Bulgaria; 3 Department of Medical Biology, Medical University—Plovdiv, 4000 Plovdiv, Bulgaria

**Keywords:** Chlorhexidine, APRF membrane, vitality, PDL cells

## Abstract

Introduction: Chlorhexidine (CHX) has been used for some time in clinical practice as a local antiseptic agent with excellent efficacy. The combination of CHX with APRF (Advanced-platelet rich fibrin) membrane has the potential to stimulate tissue regeneration and to provide a bactericidal effect. We hypothesize that this may reduce the rate of infections development and protect cell viability. Aim: The aim of this study was two-fold—to create a stable APRF membrane treated with different concentrations of CHX (0.01% and 0.02%) and to monitor its effect on the viability of PDL cells in vitro. This benefits the introduction of a new protocol for APRF membrane production -CHX-PRF and enriches the available evidence on the effect of this antiseptic agent on PDL (Periodontal ligament) cells. Materials and methods: APRF membranes were prepared by the addition of two concentrations (0.01% and 0.02%) of CHX. Membranes without the antiseptic were also prepared and used as control samples. PDL cells were cultivated on the membranes for 72 h. Cell number and vitality were examined by fluorescent cell viability assays. Results: Our results demonstrated that a concentration of 0.01% CHX allowed the production of a stable APRF membrane. This concentration slightly reduced the viability of PDL cells to 96.7%, but significantly decreased the average number of cells attached to the membrane—149 ± 16.5 cells/field compared to controls −336 ± 26.9 cells/field. APRF-CHX 0.02% membranes were unstable, indicating a dose-dependent cytotoxic effect of CHX. Conclusions: The introduced novel protocol leads to the production of a new type of APRF membrane—CHX-PRF. The incorporation of an antiseptic into the APRF membrane can improve its bactericidal activity and might serve as an important step for the prevention of postoperative infections.

## 1. Introduction

Chlorhexidine (CHX) has long been used in clinical practice as a local antiseptic agent with excellent efficacy [1]. It is available in various solutions for preoperative cleaning of the skin, prior to surgical incisions, in postoperative dressings, or by direct application in the oral cavity. It can be vital in the postoperative care of immunocompromised patients. Therefore, it is important to evaluate its biological effects and to determine the most appropriate concentrations for its use. In vitro (fibroblasts, endothelial cells, osteoblasts, etc.) and in vivo studies have shown conflicting data on the safety and efficacy of CHX [2,3,4,5,6,7,8,9,10,11,12]. The information on its cytotoxic effect on cell proliferation is scarce and largely unexplored. Several studies have reported dose-dependent cytotoxicity, demonstrated in both clinical and laboratory in vitro studies [6,13,14,15]. Other evidence supports its safety and reliability [16,17].

The platelet-rich-fibrin (PRF) is accepted as a second-generation blood derived product without the addition of anticoagulants. There are two different types of PRF: leukocyte-PRF (L-PRF) and Advanced-PRF (A-PRF) [18]. Due to the absence of anticoagulants, activation of platelets is initiated, which leads to the completion of the coagulation cascade [19]. The fibrin captures platelets and leukocytes. This fact is associated with the therapeutic potential of the membrane [20]. Recently, the use of PRF in the field of dentistry and oral surgery is increasing, especially in regenerative dentistry, periodontal and mucogingival surgery, sinus lift procedures, soft and bone tissue augmentation, infectious and drug-induced inflammations, post-extraction bleeding control, etc. [19,20,21,22,23,24,25,26,27,28,29]. However, its use is still limited, due to the sophisticated protocol and some unsuccessful preparation results in the past [30,31,32,33].

This autologous fibrin matrix demonstrates a potential to stimulate cell adhesion, proliferation [34], and differentiation [35] in vitro. Dohan et al. [21,35] reported that PRF has immunological properties and contains cytokines (released by white blood cells) that can induce angiogenesis and anti-inflammatory responses, which contribute to tissue repair [34]. According to our knowledge, there is no data in the scientific literature, to describe the treatment of PRF membrane with CHX and asses of the viability of the PDL cell line in these conditions.

Our study aimed to create a stable APRF membrane treated with specific concentration of CHX (0.01%), to monitor its effect on cell viability in vitro, and eventually to propose a novel protocol for the preparation of a genuinely new type of PRF—CHX-PRF. Since proven not disruptive to cell vitality in vitro, APRF membranes containing CHX should be further investigated for their potential in vivo effects on oral soft and hard tissues.

The innovation of the study consists of the preparation of a APRF membrane, enriched with the antiseptic agent CHX. Thus, a boost to tissue regeneration and a release of growth factors from the APRF membrane is ensured. We suggest that this may reduce the probability of infections and protect cell viability, since we managed to prove it is sustainable.

## 2. Materials and Methods

### 2.1. Chlorhexidine Solution

CHX (without additives) (Gluco-Chex 2% CERKAMED Medical Company, Stalowa Wola, Poland) was prepared as 0.01% and 0.02% aqueous solution.

### 2.2. APRF Membranes

Twenty-one healthy volunteers aged from 20 to 35 years from the Bulgarian cohort participated in the study. They all matched the inclusion criteria: no acute infections or systemic comorbidities. The exclusion criteria included patients on immunosuppressive therapy, with clotting disorders, on anti-coagulation and platelet anti-aggregation therapy. All of them signed a written informed consent for vein puncture in accordance with the instructions of the Ethics Committee at Medical University of Plovdiv (Protocol № 1746/19.07.2019) Blood samples were collected in 4 vacutainer eprouvettes of 10 mL. In two of the eprouvettes 0.2 mL of 0.01% CHX and 0.02% CHX, respectively, were injected prior to the blood drawing, and they were used for the preparation of the new CHX-PRF. One sample was used for the preparation of APRF without CHX (“APRF+” according to protocol Choukroun) and the fourth was used for routine laboratory hematology tests (Vacuette tube 10 mL K2EDTA) (Table 1).

**Figure 1:**
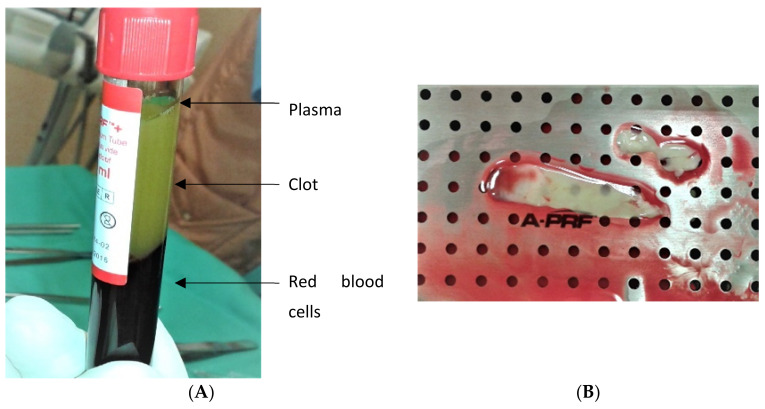
APRF membrane preparation (**A**) eprouvette after centrifugation with three different layers (red blood cell clot, platelet rich fibrin clot and liquid plasma layer); (**B**) APRF box that transforms the clot into a membrane.

The blood required for preparation of the APRF membrane and the two CHX-PRF membranes was then centrifuged in a specific device (Process for PRF Duo, CPM pharma, Gondomar, Portugal), according to the manufacturer’s low speed protocol—1300 rpm for 8 min. After centrifugation, three different layers were formed in each eprouvette (APRF + Choukroun glass eprouvette) from bottom to top as follows: red blood cell clot, platelet rich fibrin clot and liquid plasma layer (Figure 1A)

The fibrin clot was then held with anatomical tweezers and separated from the other two layers with surgical scissors. Afterwards, it was placed in a PRF box (Figure 1B) and pressed between two flat metal plates which transformed it into a membrane. The membrane was cut into even pieces, 8 mm long and placed in sterile 24-well polystyrene cell culture plates.

Two types of control samples were also used—without CXH (PDL cells without APRF membrane and APRF membranes without PDL cells), in order to examine morphological changes in the cells and in the membrane.

### 2.3. PDL Cellular Line

The PDL (Periodontal ligament) cellular line was kindly provided by Prof. Draganov as a model system for studying the effect of CHX and APRF membranes. The line has been obtained by transforming cells isolated from patients undergoing orthodontic treatment. Lentiviral gene transfer of human telomerase-reverse transcriptase (hTERT) has been performed. HTERT-expressing PDL cells showed similar morphology and population doubling time, but an extended lifespan compared to the primary cells [36]. This type of cells has a significant role in the healing of wounds and implantation sites and is a promising tool for research in the field of periodontics. The PDL line was cultivated in Dulbecco Eagle’s medium (DMEM) supplemented with 10% fetal bovine plasma (FCS), 10% FBS, 1% penicillin/streptomycin. The cells were grown at 37 °C for 72 h in an incubator (Panasonik MCO-18ACUV-PE) with 5% CO_2_ and high humidity. Cells were detached with 0.05% trypsin/EDTA solution and subcultured at a density of 1.10^5^ cells in 24 well cell on pre-prepared APRF membranes culture plates (Costar 24-well Clear TC-treated Multiple Well Plates, Costar 24-well Clear TC-treated Multiple Well Plates, no 14831 (Corning, NY, USA).

### 2.4. Cell Viability Assays

#### 2.4.1. Detection of live PDL Cells via Electronic Microscopy

Three different APRF membranes were used in the experiment—one APRF membrane and two from the newly proposed CHX-PRF membranes, created with the addition of 0.01% and 0.02% CHX. Membranes without CHX were examined as a negative control. They were placed in 24-well plates. 1.10^5^ PDL cells were added to in each well and incubated for 72 h. The cells were washed with PBS and then incubated with fluorescent dyes—Calcein-AM (CA) (Sigma-Aldrich Cat. №56496, Calcein-AM (CA) Sigma-Aldrich no 56496, (Merck SA, Darmstadt, Germany). and propidium iodide (PI) (Sigma-Aldrich Cat. №P4170). The fluorescent dye solutions were prepared according to the manufacturer’s established protocol. After incubation for 10 min at 37 °C in dark environment, the samples were rinsed twice in PBS and visualized with a fluorescence microscope Nikon eclipse no Ni-U931769 (Nikon, Amstelveen, The Netherlands) Nikon eclipse TS 100 no 450999 (Nikon) with excitation/emission at 465/495 nm for CA-green florescence and 550/25 nm for PI-red florescence. The observed image was captured with a photo documentation system (Nikon). For each sample four consecutive microscopic fields were examined at 100× magnification. Cells were counted per field and the average value was calculated for each sample. The results were confirmed by two independent examiners. The percentage of living cells was calculated by the formula:

Cell viability = 100% × live cells/(live cells + dead cell).

**Figure 2:**
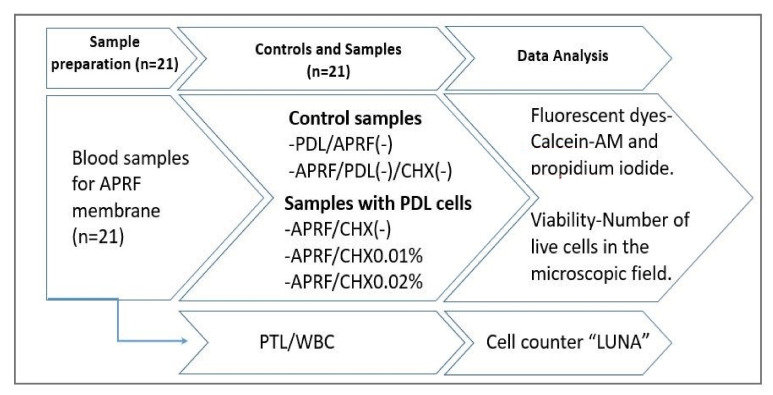
Design of the experimental work, presenting the exact sequence of the workflow.

The experimental workflow is presented on Figure 2.

#### 2.4.2. Detection of Number and Viability of Platelets (PLT) and White Blood Cells (WBC) Included in APRF Membranes

Plasma was collected before and after membrane pressure. On a cell counter “LUNA” (Logos Biosystems, Anyang, South Korea), the number and viability of platelets (PLT) and white blood cells (WBC) in APRF membranes were measured. Ten μL of the plasma obtained from the stable fibrin membranes with and without CHX was mixed with 10 μL of 0.4% trypan blue solution for cell counting. The same procedure was repeated for the plasma obtained after pressing the APRF membranes. The number of PLT and WBC present in the membrane was calculated by the difference between the number of cells in the plasma before and after pressure.

### 2.5. Statistical Analysis

All experiments were carried out in independent triplicates.

Data pre-processing was performed with MS Office Excel. Statistical analyses were performed using GraphPad Prism v.8.0.1. Data distribution was checked for normality by the Shapiro–Wilk test and by visual inspection of the QQ-plots. Differences between normally distributed variables were evaluated for significance using a Welch’s *t*-test for independent samples or a paired *t*-test for dependent samples. For non-normal data, the non-parametric Wilcoxon–Man–Whitney assessment was preferred. The significance threshold was set at a *p*-value < 0.05.

## 3. Results

This study introduced a novel protocol for the production of a new type of APRF membranes—CHX-PRF. In this type of APRF membrane we combined the antiseptic properties of CHX and APRF membrane’s ability to increase the healing process. This combination has the potential to enhance postoperative care by reducing infections in the days and weeks after the surgical procedure. For the preparation of the membranes, 21 healthy volunteers (11 females and 10 males) (Supplementary Figure S1) with a normal range of complete blood count (CBC) were selected (Table 2). Normal blood count values were important to verify that the addition of CHX is the only factor leading to differences between the membranes. There was no statistically significant difference in platelet counts among the participants in the study.

### 3.1. Evaluation of Viability, Morphology, and Mean Number of Living PDL Cells Grown on APRF Membranes

PDL cells grown on the surface of the APRF membrane without CHX (APRF/CHX (-)) showed increased vitality to 98.8% after incubation for 72 h. CHX APRF 0.01% membranes (APRF/CHX0.01%) slightly decreased the vitality of PDL cells to 96.7%. On the other hand, CHX APRF 0.02% membranes (APRF/CHX0.02%) were unstable and the experiment with them ended at an early stage. This result led to the selection of 0.01% CHX concentration as the most appropriate one for CHX-PRF preparation.

**Figure 3:**
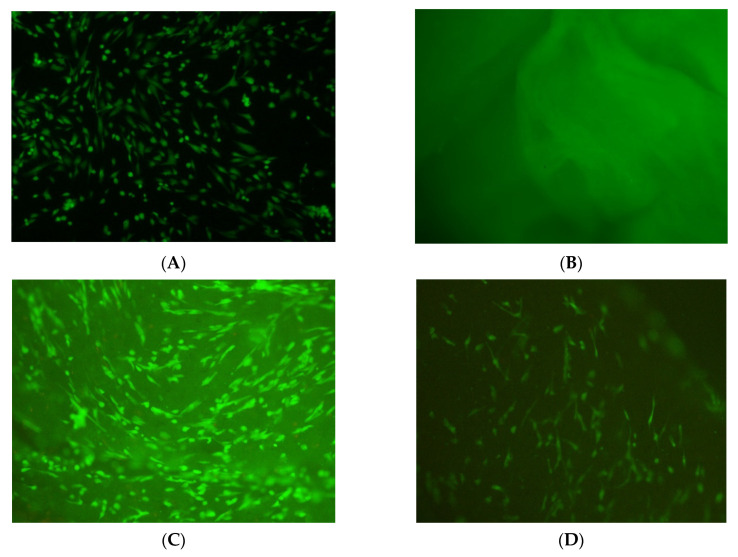
Fluorescence microscopy images of PDL cells and APRF membranes after staining with Calcein-AM (CA) and propidium iodide (PI) at 100× magnification, scale bar 200 µm: green color—staining with CA indicating living cells and red color with PI showing necrotic cells. (**A**) PDL cells without membrane (PDL/APRF (-)); (**B**) APRF membranes without CHX and without PDL cells (APRF/PDL(-)/CHX (-)); (**C**) PDL cells on a membrane without CHX (APRF/CHX (-)); (**D**) PDL cells on a membrane with 0.01% (APRF/CHX- 0.01%).

The green staining of the cells with the CA dye reflects their viability. The lack of red color with the PI staining proves that there are no necrotic cells (Figure 3). No differences were found in the morphology of PDL cells between controls (PDL/APRF (-)) and those incubated on APRF/CHX (-) or APRF/CHX 0.01%. The three groups (PDL/APRF(-), APRF/CHX(-) and APRF/CHX0.01%) showed PDL-specific spindle-shaped fibroblast morphology.

**Figure 4:**
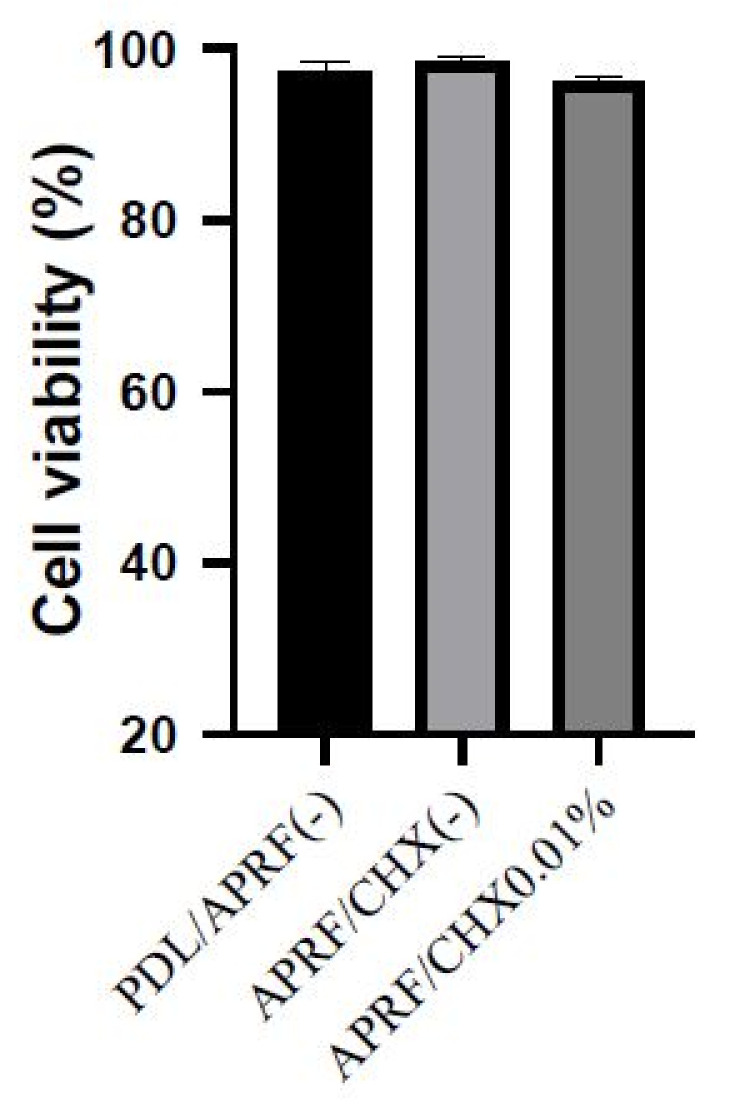
Evaluation of viability of PDL cells without membrane (PDL/APRF (-)), PDL cells cultured on APRF membrane without CHX (APRF/CHX (-)) and PDL cells on a membrane with 0.01% CHX (APRF/CHX0.01%). In all three groups, living cells are abundant, with the percentage over 95%. The fact suggests that the novel type of membrane is not inferior to the others, in terms of cell viability.

**Figure 5:**
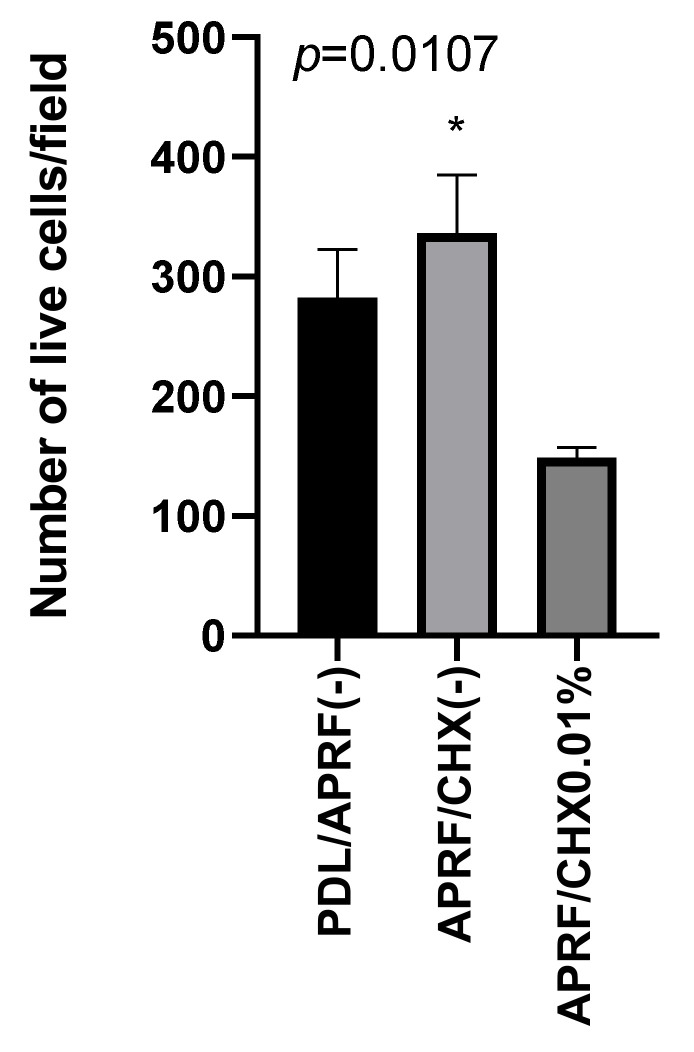
Number of live cells per microscope (fluorescence microscopy) field of view in PDL cells without membrane (PDL/APRF (-)), PDL cells cultured on APRF membrane without CHX (APRF/CHX (-)) and PDL cells on a membrane with 0.01% CHX (APRF/CHX0.01%). It demonstrates the reduced number of cells within the CHX treated membrane.

The total number of living PDL cells on APRF/CHX (-) showed a vitality of 98.8% (Figure 4). This is close to that of PDL/APRF (-)—98.6%, but the average number of cells per microscope field of view was significantly higher on APRF/CHX (-)—336 ± 26.9 cells/field, while in PDL/APRF (-) it was 282 ± 20.9 cells/field. A reduction of the number of cells on APRF/CHX0.01% (149 ± 16.5 cells/field) compared to APRF/CHX (-) (336 ± 26.9 cells/field) was observed (Figure 5). The average number of cells on APRF/CHX0.01% at magnification 100× was 149 ± 16.5 cells/field in a microscopic field.

### 3.2. Number and Viability of PLT and WBC Present in APRF Membranes

Due to the inability to directly determine the number of PLT and WBC in the APRF clot with and without CHX, we measured the number and viability of these cells before and after pressing the membrane (Table 3). Thus, blood cells (present in the APRF membrane) can be indirectly evaluated.

**Figure 6:**
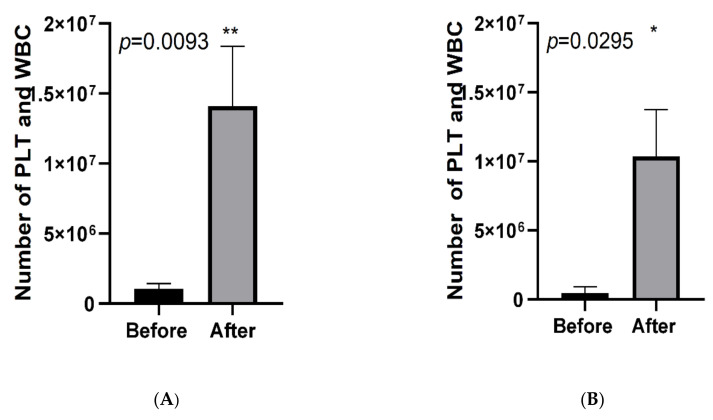
Number of PLT and WBC before and after pressing APRF membranes.

After the paired Wilcoxon test for comparison of the number of living cells on the APRF membrane before and after the pressing, a statistically significant difference was observed with W = 107 and *p*-value = 0.0093 and a higher number of cells in the plasma after the pressing was detected (Figure 6A). Findings were similar for the 0.01% CHX with a statistically significant difference—W = 69 and *p*-value = 0.0295 (Figure 6B).

**Figure 7:**
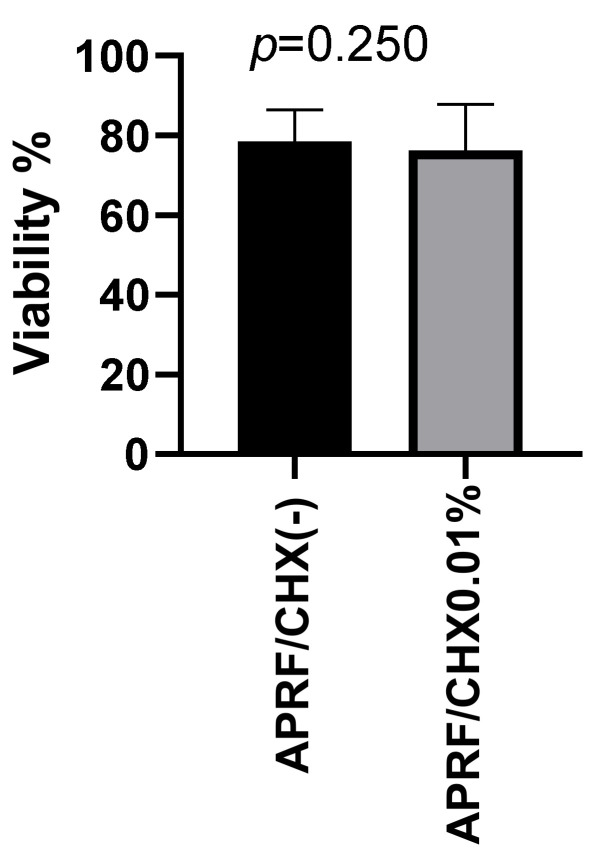
Viability of the PLT and WBC in APRF membranes.

The comparison in cell vitality of PLT and WBC measured by cell counter did not show a statistical difference for APRF/CHX (-) and APRF/CHX0.01%. The average vitality of the cells from APRF/CHX (-) was 78.5%, and for those with APRF/CHX 0.01–76.2%. There was no significant difference in the viability of the PLT and WBC found in both membranes-W = −34, *p* = 0.250 (Figure 7), which also supports the results obtained with the fluorescent staining. This suggests that the low concentration of the antiseptic did not have a significant effect on cell survival in vitro.

## 4. Discussion

Both Chlorhexidine and PRF are widely used in dentistry in general and in oral surgery in particular. Although various options for postoperative infection control and healing promotion exist, most of them have side effects and there is room for innovation. The combination and possible in vivo cumulation of the positive properties of CHX and PRF can be useful for patient care, especially in immunocompromised patients, ones with moderate or severe diabetes, patients that demonstrate impaired bone metabolism, etc.

Investigation on periodontal cellular lines only is on the one hand limitation, given the variety of cell types that are present in the oral cavity, but is on the other hand pivotal, because periodontal regeneration is an important procedure in the field of dentistry.

The present in vitro study evaluates the vitality of the PDL cell line for 72 h on an APRF/CHX0.01%. The results revealed a slight inhibition of viability (96.7% living cells), but a more significant reduction in the average number of cells per microscope field of view attached to the membrane (149 ± 16.5 cells/field) compared to APRF/CHX (-) (336 ± 26.9 cells/field). We consider this a potential result from the slow release of CHX from the membrane, which may allow its antiseptic activity. As a comparison, APRF/CHX (-) showed cell vitality of 98.8%. This membrane serves as a substrate that might stimulate cell division in wound healing and regeneration. The relationship between PLT in APRF and the mitogens released is shown by Gruber et al. (2003) [37]. These cells are known to contain growth factors such as PDGF, TGF-b, and IGF-I, which can activate cell proliferation [19,38]. The PDL cells are in a microenvironment composed of an extracellular matrix rich in growth factors [39], which explains the observed results in CHX-free membranes. We also found that the APRF/CHX0.01% was stable and had no significant effect on cell survival in vitro. A group of results by some authors support our data on PLT and WBC detected in the APRF. They also reported varying degrees of cytotoxicity and inhibited adhesion depending on the time and concentration of CHX [6,7,40,41,42,43,44].

Madhusudanan et al. (2020) demonstrated that treatment with CHX may cause drug-induced damage to fibroblasts [45]. Exposure of cultured fibroblasts, myoblasts, and osteoblasts to 0.02% CHX or higher concentration of the antiseptic resulted in impaired cell function [2].

One of the limitations of this study is that in vitro cell cultures do not fully reflect the cell profile in the oral cavity, which consists of various different cell types. Studies have shown that in vivo tissues generally have a higher tolerance to antiseptic solutions than in vitro cell cultures. It could be assumed that the decreased number of live cells per microscope field of view will affect wound healing in vivo [14,40].

Despite these limitations, we present original evidence, which shows that CHX at concentrations of 0.01% can be used for the preparation of stable APRF membranes and affects the vitality of PDL cell cultures insignificantly.

APRF membranes, prepared with the addition of CHX, show great potential and might prove beneficial as a material of choice in a large variety of soft and hard tissue procedures in dentistry, oral surgery and dental implantology such as periodontal surgical procedures in terms of chronic inflammation, tooth extraction in terms of chronic or acute inflammatory processes and especially bisphosphonate medications intake, immediate implant placement, surgical vertical and horizontal bone augmentation, periodontal and preprosthetic soft tissue grafting, etc.

## 5. Conclusions

Our study proposed a novel protocol for the production of a genuinely new type of APRF membrane—CHX-PRF. Our results showed that a concentration of 0.01% CHX allowed the production of a stable APRF membrane and had a minor effect on the number of PLT and WBC present in the APRF clot (76.2% vital cells). This CHX concentration did not remarkably reduce the growth of the PDL cell line (96.7% vital cells). At 0.01% CHX concentration, the average number of living cells per microscope field of view decreased in comparison to APRF/CHX (-) (149 ± 16.5 cells/field). Different concentrations of CHX and their effect on cell viability were tested prior this experiment. The data we obtained showed that low concentrations of CHX reduce its adverse effects, as higher doses are cytotoxic and disrupt the formation of a stable APRF clot. As a conclusion, this novel type of membranes definitely indicates further investigation both in vitro and in vivo.

## Figures and Tables

**Table 1:** Abbreviations used in the experiment.

Control Samples	
**PDL/APRF (-)**	PDL cells without APRF membrane
APRF/PDL (-)/CHX (-)	APRF without CXH, without PDL
**Samples with PDL**	
APRF/CHX (-)	PDL cells on a membrane without CHX
APRF/CHX 0.01%	PDL cells on a membrane with 0.01% CHX
APRF/CHX 0.02%	PDL cells on a membrane with 0.02% CHX

**Table 2:** Average parameters of the complete blood count (*n* = 21). The values of significance for us are WBC, PLT and HCT, because serious deviations in these values could possibly affect the normal formation of the fibrin clot.

CBC	Average	STDEV	Ref. Range	Unit
WBC	6.73	1.99	3.5–10	10^9^/L
RBC	5.00	0.68	3.5–5.5	10^12^/L
PLT	260.89	69.37	140–400	10^9^/L
HGB	148.42	14.79	110–160	g/L
HCT	0.43	0.04	0.35–0.54	L/L
MCV	86.95	4.42	82–95	fL
MCH	29.96	1.97	27–32	pg
MCHC	344.26	9.82	300–340	g/L
RDW-Cv	13.74	0.64	11–16	%
RDW-Sd	42.65	1.98	35–56	fL
MPV	10.30	1.13	6.5–12	fL
PDW	12.46	2.20	9–17	fL
PCT	0.27	0.07	0.108–0.282	%
P-LCR	28.51	7.76	11–45	%
P-LCC	73.26	23.68	30–90	10^9^/L

**Table 3:** Number of PLT and WBC present in APRF membrane.

APRF Membrane	Before Pressing	After Pressing	After-Before Pressing APRF Membrane
APRF/CHX (-)	1.05 × 10^6^	1.41 × 10^7^	1.3 × 10^7^
APRF/CHX 0.01%	4.50 × 10^5^	1.04 × 10^7^	9.90 × 10^6^

## Data Availability

The data presented in this study are available in the article.

## Supplementary Materials

The following supporting information can be downloaded at: https://www.mdpi.com/article/10.3390/jfb13040226/s1, Figure S1: No statistically significant difference between men (10) and women (11) in platelet counts.
