# Supplementary material for: Effect on Cellular Vitality In Vitro of Novel APRF-Chlorhexidine Treated Membranes
Source: J Funct Biomater. 2022 Nov 7;13(4):226. doi: 10.3390/jfb13040226 (PMC9680238; doi:10.3390/jfb13040226)
Supplement: Supplementary file 1 [file jfb-13-00226-s001.zip › jfb-1965549-supplementary.pdf]

Journal of
Functional Biomaterials

MDPI

Article

Effect on Cellular Vitality In Vitro of Novel APRF-Chlorhexidine Treated Membranes

Tasho Gavrailov [1,*], Ivan Chenchev [1,2], Maria Gevezova [2,3], Milena Draganova [2,3] and Victoria Sarafian [2,3]

1 Department of Oral surgery, Medical University—Plovdiv, 4000 Plovdiv, Bulgaria
2 Research Institute at MU-Plovdiv, 4000, Plovdiv, Bulgaria
3 Department of Medical Biology, Medical University—Plovdiv, 4000 Plovdiv, Bulgaria
* Correspondence: tasho.gavrailov@mu-plovdiv.bg

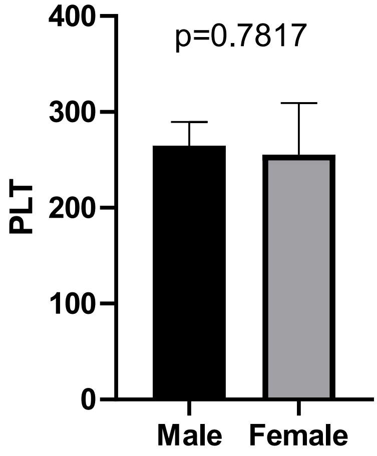

Supplementary Figure S1. No statistically significant difference between men (10) and women (11) in platelet counts.
